# Late-stage Attentional Bias towards Food Cues Varies According to Weight Status

**DOI:** 10.18103/mra.v11i6.3918

**Published:** 2023-05-09

**Authors:** Nicholas B. Wheeler, Jordan A. Colella, Robert E. Anderson, Kylie F. McFee, Kyle D. Flack

**Affiliations:** 1 Department of Health and Clinical Sciences, College of Health Sciences, University of Kentucky, Lexington, KY; 2 Department of Dietetics and Human Nutrition, College of Agriculture, University of Kentucky, Lexington, KY

**Keywords:** Attentional Bias, Obesity, Environmental Salience, Food Reinforcement, Visual Probe Procedure, Fixation time

## Abstract

The Current food environment has become increasingly obesogenic, with rates of obesity and related conditions continually rising. Advertisements for energy-dense foods are abundant and promote unhealthy eating behaviors by capitalizing on one’s attentional bias towards food cues, a cognitive process resulting from the sensitization of highly reinforcing food. A heightened awareness towards food cues may promote overconsumption of energy-dense foods. The current study employed novel eye-tracking methodology to capture sustained, or late-stage, attentional bias towards food cues. Late-stage attentional bias is the aspect of attentional bias under conscious control and likely more prone to modification compared to initial/ early-stage attentional bias, which reflects automatic processes. The present study hypothesized late-stage attentional bias towards food cues is greater among individuals classified as overweight/obese than those classified as normal weight. Thirty (30) participants classified as overweight/obese (BMI ≥25) and 47 classified as normal weight (BMI <25) were assessed for late-stage attentional bias towards food cues, conceptualized as the percentage of time fixated on food cues when both food and neutral images were presented during a food-specific visual probe procedure task. Percentage of time fixated on food cues was 51.25 ± 1.27 (mean + SE) among individuals classified as overweight to obese while those classified as normal weight had a percent fixation of 47.26 ± 0.87 (P=0.03). In conclusion, individuals classified as overweight to obese have greater late-stage attentional bias towards food cues. This establishes an important factor influencing energy intake that may be modified in future clinical trials.

## I. Introduction

Obesity prevalence in the United States has steadily risen from 8% in the early 1980s to 22% by 2005^1^. Today, over 70% of the American population is classified as overweight or obese^2,3^. Understanding contributing factors to this endemic could be essential for reversing these trends and improving the health of many.

Overconsumption of energy dense foods is a main contributing factor in the rising number of individuals classified as overweight to obese^1,4,5^. Inherent biases individuals have related to sensory or emotional feelings towards these foods is known to play a role in such overconsumption^6^. Food advertisements commonly found throughout popular media target these sensory and emotional feelings, promoting an environmental salience towards these foods, increasing their attractiveness^7^. Energy-dense, palatable foods are the most abundantly advertised foods, especially those targeting children and adolescents^8,9^. These advertisements have been linked to the overconsumption of energy dense, nutrient poor food and unhealthy BMI^10–12^. These correlations can be explained by the heightened responsiveness to environmental food cues individuals classified as overweight to obese can display, an area that has been under study as early as the 1970’s with Rodin and Slochower’s seminal work^13,14^. The term “attentional bias” has since been coined to convey this heightened responsiveness, specifically referring to a heightened attention toward a particular stimulus. Attentional bias towards addictive stimuli are well documented 15–18 supporting the incentive-sensitization hypothesis, which posits rewarding stimuli capture attention and induce cravings^19^. Following up on Rodin and Slochower’s initial work, attentional bias research has extended to eating behaviors, employing behavioral tasks such as a food-specific Stroop test or Dot-Probe tasks to measure reaction time and accuracy in identifying colors of food and neutral words. For these tasks, reaction time and accuracy become surrogate measures for attentional bias. Indeed, several trials have demonstrated a link between weight status and these measures of attentional bias, whereas individuals classified as overweight or obese have greater attentional bias towards food cues than normal weight controls^20–23^.

More recently, eye-tracking technology has come to the forefront of attentional bias research, allowing for the noninvasive assessment of eye movements as individuals examine visual stimuli. Eye movements are directly observable and an ecologically valid measurement of the visual attention process, measuring the tendency to automatically devote attention to specific stimuli^24,25^. This data provides detailed information into how individuals respond to visual stimuli in real time. Unlike the aforementioned behavioral indices of attentional bias (reaction time, accuracy), eye tracking provides a direct measure of attentional processing and can differentiate between early and late stages of attentional processing. The direction of an initial fixation is thought to assess automatic processes related to the initial salience of the visual stimuli whereas total fixation time (time spent looking at one visual stimuli over another) is considered to assess late stages of attentional processing that are under coconscious control^26^. Many of these studies have utilized a visual dot-probe task in conjecture with eye tracking, where two images are presented on a computer screen (one food and one non food/neutral) for a certain amount of time before a probe appears in place of one of the images. Participants are required to respond to the probe by selecting the corresponding computer key as quickly as they can indicating if the probe appears on the right or left side of the screen. In this way, both visual eye tracking data (assessed when looking at images before probe appears) and reaction time (time to react to probe) can be assessed as measures of attentional bias^25,27–29^. These studies all report differences in attentional bias between individuals classified as overweight to obese when compared to normal weight controls, although the parameters and exact indices seem to vary. Specifically, Werthmann et al. demonstrated only initial fixation direction and the time spent in that initial fixation differed between groups, indicating automatic processing is primarily different between weight status group^25^. Both Nijs et al. and Castellanos et al. additionally assessed the effects of fed and fasted states on attentional bias between weight status groups. Nijs et al. demonstrated no difference between groups or fed/fasted conditions in eye tracking data, whereas all participants demonstrated a greater attentional bias towards food. Interestingly, when looking at reaction time, this study demonstrated normal-weight individuals responded faster to the visual probe than the overweight/obese group^29^. Castellanos et al. demonstrated all participants had an elevated attentional bias while fasted, while only the overweight/obese group demonstrated greater attentional bias towards food cues in the fed condition. In contrast to Werthmann et al., these results applied to both initial fixation direction and total fixation time^28^. Graham et al. took a different twist, assessing the interaction between different types of food (low energy, high-energy sweet, high-energy savory) and weight status. Interestingly, total fixation time did not differ between groups, while initial fixation direction was greater towards low-energy foods in the overweight/obese group compared to control^27^.

Employing these adjustments to the Dot-Probe task with eye tracking, the current study sought to demonstrate differences in attentional bias towards food cues (specifically late-stage attentional bias, conceptualized as percent fixating time) between individuals classified as overweight to obese (BMI≥25) and those who are classified as normal weight (BMI <25). We hypothesized individuals classified as overweight or obese would spend a greater percentage of time fixated on food cues than those classified as normal weight, indicating they have greater late-stage attentional processing, which is under coconscious control, as opposed to initial fixation direction that is automatic^25^.

## II. Methods

### 2.1 Participants

Participants classified as normal weight, BMI <25 (N=47, 24 female) and those classified as overweight to obese, BMI ≥25 (N=30, 14 female) were recruited using flyers and email advertisements. Recruitment occurred between April and November 2021 and was approved by the University of Kentucky IRB (protocol # 69120). Participants could not be tobacco users, pregnant, or nursing. Individuals were also required to be at least 18 years old. The sample of participants classified as overweight to obese were recruited from a separate study that included baseline assessments for attentional bias and body composition^30^. The inclusion/exclusion criteria remained the same for both studies, except for weight status, with only healthy individuals absent of any chronic condition (diabetes, heart disease, cancer) included.

### 2.2 Measures

#### 2.2.1 Height and Weight

Height was measured in triplicate to the nearest 0.1 cm using a stadiometer (Seca; Chino, CA). Body weight was measured using a calibrated digital scale connected to the BodPod (Cosmed, USA) to the nearest 0.01 kg. Measures were completed with participants wearing spandex shorts and sports bra (females) or no shirt (males) as required for the BodPod assessment (below). BMI was calculated during the screening and enrollment visit to ensure participants qualified for the study.

#### 2.2.2 Body Composition

Kg of body fat and fat-free mass were determined via air displacement plethysmography (BodPod, Cosmed USA) and percent body fat calculated. Participants were assessed prior to completing the attentional bias task and were at least 3 hours post-prandial. The BodPod is a reliable and valid assessment tool for body composition in adults, offering a quick and noninvasive assessment of body composition^31^. The Siri or Schutte density model was used, depending on race, to convert body density to percent body fat^32,33^. Thoracic gas volume was measured according to manufacturer’s recommendations.

#### 2.2.3 Attentional Bias

The current study employed similar eye-tracking procedures as previous studies noted with a few important adjustments. First, we utilized a 3,000ms presentation time for the pictures of food cues (before being replaced by the visual probe). Of the prior studies mentioned, Castellanos et al.’s 2,000 ms presentation time was the longest, while others typically use 500 or 1,000ms, following prior eye-tracking addiction research that traditionally utilizes a 1,000ms presentation time^15–18^. The issue, we believe, in barrowing from addictive research in this scenario, is that food is much less reinforcing to most people compared to the reinforcing value of drugs of abuse among those with an addictive disorder^34,35^. Therefore, individuals with an addictive disorder will be much more sensitive towards drug-related stimuli than a healthy individual is towards food. Prior to our eye tracking studies, we piloted this task and observed great variability among 5 test subjects using the 1,000ms presentation time (within subject SD range 0–50.1) and for the 2,000ms presentation time (SD range 11.5 to 43.9). The 3,000ms presentation time yielded a within-subject SD range of 14.9 to 21.8 and therefore was chosen for this study. We believe this may be the reason some of these prior studies did not observe a difference between groups or conditions in total fixation time. We also improved upon prior studies by developing a novel metric to assess total fixation time, or late-stage attentional bias. This new metric, mean percentage of time fixated on food cues, or *percent fixation time*, is calculated by dividing the fixation time for the food cue by the fixation time for the food + neutral cue. This is done for each individual trial and then averaged across the entire test, providing an average % fixation time. This is an improvement over the traditional total fixation time as some trials may result in 0ms of fixation time for either image (in the event a participant blinks or looks away and their eyes were not recaptured quick enough). If a participant has even one of these trials, their total fixation time may look greatly reduced compared to a participant who did not have any such trials. By expressing fixation time as a percentage (and removing the trials that did not register any fixation time) we avoid this potential issue. Lastly, the current study assessed individuals who were neither satiated nor fasted, assessing every participant in the afternoon between lunch and dinner who reported to be only moderately hungry. This may represent a more typical, real-world scenario, where someone is not extremely hungry nor extremely full.

Attentional bias was assessed by the visual probe procedure adapted for food cues^36–38^. Ten images of energy dense foods (critical task stimuli) were paired with ten images of non-food (neutral) stimuli that included nature images, furniture, and buildings. Other pairs included two neutral (non-food) images used as filler trials. All image pairs were matched for color and complexity, had dimensions of 680×491 pixels with a 96 dpi. Pairs were presented one at a time in random order. Image pairs were shown for a total of 3,000ms after which a yellow dot appeared in place of one of the images. Participants were instructed to right click the computer mouse if the yellow dot appeared on the right side of the screen and left click if the yellow circle appeared on the left of the screen. Participants were positioned so that their eyes were 65–75cm away from the screen. Calibrations were performed to ensure that the participants’ eyes were being tracked properly before each trial. Each pair of images (both those containing food images and those not) were shown twice. The orientation of the images were flipped so neither food nor neutral image was shown on the same side of the screen twice. There was a total of 80 trials (pairs) shown to each participant. Half (40) of the trials contained food cues and the other 40 pairs contained only non-food, filler, trials. The primary outcome was time in ms fixated on food and neutral images, which was used to calculate the percentage of time fixated on food cues as: ms fixated on food cues / (ms fixated on food cues + ms fixated on neutral cues).

#### 2.2.4 Hunger and Satiety

Hunger and Satiety was assessed prior to the attentional bias assessment via visual analog scales (VAS) where participants dragged a slider on a computer to indicate how hungry they were on a scale of 0 “not hungry at all” to 100 “extremely hungry.” Participants were scheduled for their assessment visit between lunch and dinner and instructed to attend the experimental session 3–5 hours post prandial. This was designed so participants were neither hungry or satiated, thus assessing attentional bias under neutral conditions.

### 2.3 Statistical analysis

Repeated measures analysis of variance (ANOVA) was used to assess differences in attentional bias and demographic variables between groups (obese/overweight vs normal weight). A linear regression model using attentional bias as the dependent variable was constructed using body fat percentage and age as predictor variables. In the planned repeated measures ANOVA a sample of 30 participants has 80% power to detect an interaction which is 0.37 times the standard deviation between participants provided the correlation within participants is 0.5 or larger (effect size 0.517). Thus, our sample size of 30 and 47 participants for each group was adequate.

## III. Results

The group classified as obese/overweight was younger, had a greater BMI, and a greater body fat percentage when compared to the normal weight group (Table 1). To test if these differences influenced attentional bias for food cues, a linear regression model was constructed to determine predictors of attentional bias. This model demonstrated age was not a significant predictor of attentional bias (P=0.30) while BMI and body fat were a significant predictors (P<0.05).

**Figure 1:**
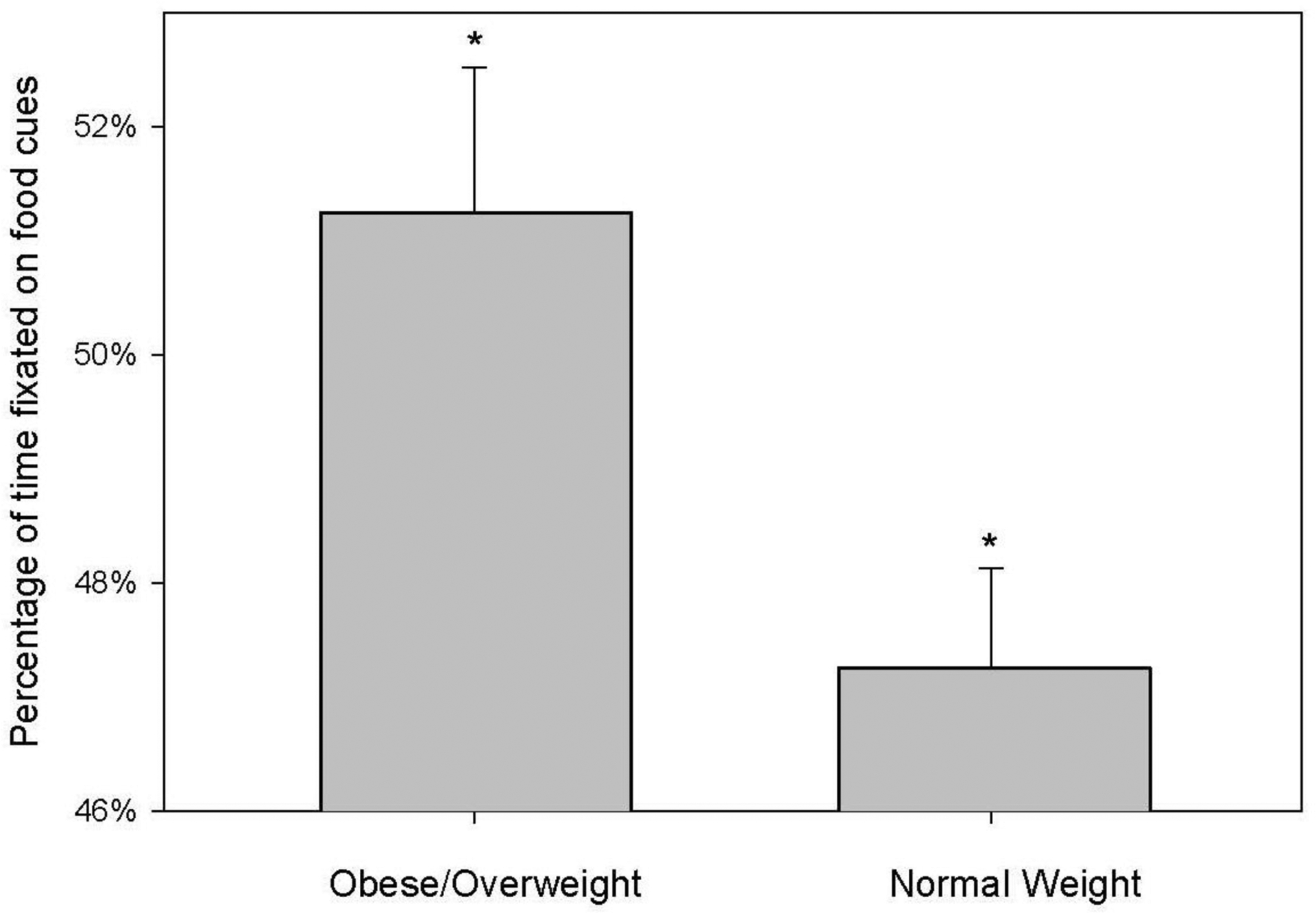
percentage of time fixated on food cues during the food-specific Dot-Probe task significantly is significantly greater among participants classified as overweight to obese (BMI≥25) than those of normal (BMI <25). *Significantly different between conditions, P=0.03

As depicted in Figure 1, there was a significant difference (P = 0.03) in percent fixation time between groups. Percent fixation time on food was 51.25 ± 1.27 among individuals classified as overweight to obese while those classified as normal weight had a percent fixation of 47.26 ± 0.87 (mean ± SE).

As expected, hunger was not correlated with attentional bias scores (r=0.13, P>0.2). This is likely due to participants were neither very hungry of very satiated when assessed, mean ± SE hunger score: 41.5 ± 5.6.

## IV. Discussion

In line with our hypothesis, the current study demonstrated individuals classified as overweight to obese have a greater late-stage attentional bias towards food cues than their normal-weight counterparts. Although a cause and effect relationship cannot be determined from the present cross-sectional study, others have linked greater energy intakes to greater attentional bias for food cues and food reinforcement^39–43^ while others have linked greater attentional bias towards food cues to future weight gain^44^. This supports the notion obesity is not creating an attentional bias towards food cues, rather, obesity is the result of an attentional bias-mediated increase in energy intake^25,41,42^.

Unlike prior studies addressing this question, attentional bias was conceptualized as the average percentage of time fixated on food cues after a 3,000ms presentation time, indicative of greater attentional processing that is under coconscious control. We chose not to focus on initial fixation direction as 1) this has been studied before and is not influenced by the novel aspects of the present study (3,000ms presentation time, % fixation time calculation) and 2) this automatic process is likely not modifiable in future training interventions.

When considering the research on attentional bias towards food cues and its relationship with eating behaviors and weight status as a whole, it is difficult to arrive at a single conclusion due to the various methodology. A review of attentional bias literature by Hendrikse et al. recognized this wide-range of methodology in conceptualizing attentional bias, such as total fixation duration towards food cues, the order of fixation (initial, latent) or the reaction time to a dot probe^20,41,42,45^. Despite the inconsistency of assessment techniques, this review concluded that there is sufficient evidence demonstrating attentional bias towards food cues is increased among individuals classified as overweight to obese^42^.

Conversely, a recent review concluded that individuals classified as overweight or obese did not differ when compared to normal weight controls in their fixation-direction or fixation-duration bias^46^. Participant characteristics such as the physiological state assessed (fasted vs. satiated), age, sex, or other pre-existing conditions may have all contributed to variability between studies to cause difficulties in identifying differences. The 3,000ms presentation time of the current trial, conceptualizing attentional bias as mean percent fixation time, and assessing individuals who were neither extremely hungry or satiated are all potential reasons we were able to identify differences in attentional bias, specifically late-stage, in our groups of normal weight vs. those classified as overweight to obese.

A possible underlying physiological response for the increased attentional bias with obesity lies in a modification of central neuronal pathways^47^. This is well documented in the drug abuse literature, where repeated exposure to the stimuli promotes a sense of wanting and an unavoidable craving^48^. Food cravings, much like drug abuse, increases the neural activity of the corticolimbic-striatal regions of the brain (motivation-reward regions) and is more pronounced among individuals classified as obese^49^. This is also supported in studies identifying a genetic component to food cue reactivity that share similarities with drug abuse^50–53^. As noted, this is likely a prime cause of over consumption and obesity, but also may pose as a barrier to weight loss as these individuals are more affected by food cues than their normal-weight counterparts. This scenario, where some individuals are more susceptible to food’s reinforcing effects due to certain genetic components of their dopamine reward system, is difficult to manage as exposure to the reinforcer (food in this case) is necessary for survival. This is an area where attentional bias training interventions could play a major role, aiding those individuals who are highly sensitive to food cues to either prevent weight gain or promote weight loss. Some attentional bias training trials are already underway and have demonstrated promising results for modifying late-stage attentional bias towards food cues^54^. The present trial, and its focus on late-stage attentional bias, is designed to provide foundational knowledge for such future attentional bias training studies- altering conscious attentional bias towards food cues to influence eating behaviors and promote obesity treatment. Future trials should focus on this aspect of attentional bias as these conscious processes governing eating behaviors is likely more relevant as we move forward towards these training studies.

Future research may also focus on whether there is an attentional bias towards low energy dense foods, such as vegetables, as most literature only considers energy dense foods. As noted, Graham et al. demonstrated that initial fixation direction was greater towards low-energy foods in the overweight/obese group compared to control^27^. A better understanding as to why this was the case is needed, if it was a product of social desirability or if the presentation time was not long enough for participants to realize what they were looking at and focus on the high-energy dense foods. Future studies using percent fixation time to conceptualize attentional bias under conscious control is needed as this aspect of attentional bias is what future attentional bias training studies can target in attempting to promote changes in eating behaviors. A limited number of these training studies have been successful in altering attentional bias towards food cues^47,55^, although it would be interesting to see if using a 3,000 ms presentation time and the percent fixation time metric would uncover additional information regarding changes in sustained fixation.

### 4.1 Limitations

The current study did not screen for eating disorders that would influence attentional bias towards food cues^56^. However, none of the participants had a BMI that would be expected with the most common eating disorder, anorexia nervosa, with the lowest BMI at 18.8 and considered normal weight. Besides body fat and BMI, the groups differed on age, although age did not predict attentional bias when included in the regression model. We also did not assess demographic factors that may be predictors of attentional bias for food cues such as food security, household income, and education.

## V. Conclusions

The primary results of the present trial supported those of previous trials which have indicated a possible link between attentional bias and weight status^20,41,42,45^. The present trial expands on these previous findings by 1: increasing the presentation time to 3,000ms and focusing on late-stage attentional bias, 2: utilizing a novel “percent fixation time” metric to better conceptualize attentional bias towards food cues, and 3: performing these tests between meals in a state where participants were neither hunger or fully satiated. Such methodological intricacies are important to consider when evaluating attentional bias for food cue research and making conclusions.

## Figures and Tables

**Table 1.** Participant characteristics. Data presented as means ± SD.

	Overweight(n=30)	Normal Weight (n=47)
Age (years)*	31.8 ± 8.53	39.9 ± 16.05
BMI (kg/m^2^)*	32.7 ± 5.20	22.6 ± 1.65
% body fat*	38.8 ± 8.08	21.6 ± 7.36

* indicates significant difference between groups (P<0.05)

BMI = Body Mass Index

## Data Availability

All data is publically available on FigShare at: https://doi.org/10.6084/m9.figshare.19104614.

## References

[1] Westerterp KR, Speakman JR. Physical activity energy expenditure has not declined since the 1980s and matches energy expenditures of wild mammals. Int J Obes (Lond). Aug 2008;32(8):1256–63. doi:10.1038/ijo.2008.74 18504442

[2] Castellanos EH, Charboneau E, Dietrich MS, Obese adults have visual attention bias for food cue images: evidence for altered reward system function. Int J Obesity. Sep 2009;33(9):1063–1073. doi:10.1038/ijo.2009.138 19621020

[3] Health, United States, 2016: With Chartbook on Long-term Trends in Health. 2017. Health, United States. 28910066

[4] Kral TV, Rolls BJ. Energy density and portion size: their independent and combined effects on energy intake. Physiol Behav. Aug 2004;82(1):131–138. doi:10.1016/j.physbeh.2004.04.063 15234601

[5] Drewnowski A Energy density, palatability, and satiety: Implications for weight control. Nutr Rev. Dec 1998;56(12):347–353. 9884582 10.1111/j.1753-4887.1998.tb01677.x

[6] Morales I, Berridge KC. Liking’ and ‘wanting’ in eating and food reward: Brain mechanisms and clinical implications. Physiol Behav. Dec 1 2020;227doi:ARTN 113152 10.1016/j.physbeh.2020.113152 PMC7655589 32846152

[7] Harris JL, Pomeranz JL, Lobstein T, Brownell KD. A crisis in the marketplace: how food marketing contributes to childhood obesity and what can be done. Annual review of public health. 2009;30:211–25. doi:10.1146/annurev.publhealth.031308.100304 18976142

[8] Pinto A, Pauzé E, Mutata R, Roy-Gagnon M-H, Potvin Kent M. Food and Beverage Advertising to Children and Adolescents on Television: A Baseline Study. Int J Environ Res Public Health. 2020;17(6):1999. doi:10.3390/ijerph17061999 32197390 PMC7142724

[9] The Impact of Food Advertising on Childhood Obesity. American Psychology Association. November 17, 2010 2010;

[10] Boyland EJ, Whalen R. Food advertising to children and its effects on diet: review of recent prevalence and impact data. Pediatr Diabetes. Aug 2015;16(5):331–7. doi:10.1111/pedi.12278 25899654

[11] Andreyeva T, Kelly IR, Harris JL. Exposure to food advertising on television: associations with children’s fast food and soft drink consumption and obesity. Econ Hum Biol. Jul 2011;9(3):221–33. doi:10.1016/j.ehb.2011.02.004 21439918

[12] McClure AC, Tanski SE, Gilbert-Diamond D, Receptivity to television fast-food restaurant marketing and obesity among U.S. youth. American journal of preventive medicine. Nov 2013;45(5):560–8. doi:10.1016/j.amepre.2013.06.011 24139768 PMC3934414

[13] Rodin J, Slochower J, Fleming B. Effects of degree of obesity, age of onset, and weight loss on responsiveness to sensory and external stimuli. J Comp Physiol Psychol. Jun 1977;91(3):586–97. doi:10.1037/h0077354 874124

[14] Rodin J, Slochower J. Externality in the nonobese: effects of environmental responsiveness on weight. J Pers Soc Psychol. Mar 1976;33(3):338–44. doi:10.1037//0022-3514.33.3.338 1271215

[15] Alcorn JL 3rd, Marks KR, Stoops WW, Rush CR, Lile JA Attentional bias to cannabis cues in cannabis users but not cocaine users. Addictive behaviors. Jan 2019;88:129–136. doi:10.1016/j.addbeh.2018.08.023 30176501 PMC6191321

[16] Marks KR, Alcorn JL 3rd, Stoops WW, Rush CR Cigarette Cue Attentional Bias in Cocaine-Smoking and Non-Cocaine-Using Cigarette Smokers. Nicotine & tobacco research : official journal of the Society for Research on Nicotine and Tobacco. Sep 2016;18(9):1915–9. doi:10.1093/ntr/ntw026 26920649 PMC4978977

[17] Marks KR, Pike E, Stoops WW, Rush CR. The magnitude of drug attentional bias is specific to substance use disorder. Psychology of addictive behaviors : journal of the Society of Psychologists in Addictive Behaviors. Sep 2015;29(3):690–5. doi:10.1037/adb0000084 25961149 PMC4586301

[18] McHugh RK, Murray HW, Hearon BA, Calkins AW, Otto MW. Attentional bias and craving in smokers: the impact of a single attentional training session. Nicotine & tobacco research : official journal of the Society for Research on Nicotine and Tobacco. Dec 2010;12(12):1261–4. doi:10.1093/ntr/ntq171 20961974

[19] Robinson TE, Berridge KC. The neural basis of drug craving: an incentive-sensitization theory of addiction. Brain research Brain research reviews. Sep-Dec 1993;18(3):247–91. 8401595 10.1016/0165-0173(93)90013-p

[20] Kemps E, Tiggemann M, Hollitt S. Biased attentional processing of food cues and modification in obese individuals. Health psychology : official journal of the Division of Health Psychology, American Psychological Association. Nov 2014;33(11):1391–401. doi:10.1037/hea0000069 24707847

[21] Braet C, Crombez G. Cognitive interference due to food cues in childhood obesity. J Clin Child Adolesc Psychol. Mar 2003;32(1):32–9. doi:10.1207/S15374424JCCP3201_04 12573930

[22] Nijs IM, Franken IH, Muris P. Food-related Stroop interference in obese and normal-weight individuals: behavioral and electrophysiological indices. Eat Behav. Dec 2010;11(4):258–65. doi:10.1016/j.eatbeh.2010.07.002 20850061

[23] Long CG, Hinton C, Gillespie NK. Selective processing of food and body size words: application of the Stroop Test with obese restrained eaters, anorexics, and normals. Int J Eat Disord. Apr 1994;15(3):279–83. doi:10.1002/1098-108x(199404)15:3<279::aideat2260150312>3.0.co;2-2 8199609

[24] Appelhans BM. Neurobehavioral inhibition of reward-driven feeding: implications for dieting and obesity. Obesity (Silver Spring). Apr 2009;17(4):640–7. doi:10.1038/oby.2008.638 19165160

[25] Werthmann J, Roefs A, Nederkoorn C, Mogg K, Bradley BP, Jansen A. Can(not) take my eyes off it: attention bias for food in overweight participants. Health psychology : official journal of the Division of Health Psychology, American Psychological Association. Sep 2011;30(5):561–9. doi:10.1037/a0024291 21767019

[26] Horsley TA, de Castro BO, Van der Schoot M. In the eye of the beholder: eye-tracking assessment of social information processing in aggressive behavior. J Abnorm Child Psychol. Jul 2010;38(5):587–99. doi:10.1007/s10802-009-9361-x 19823928 PMC2880233

[27] Graham R, Hoover A, Ceballos NA, Komogortsev O. Body mass index moderates gaze orienting biases and pupil diameter to high and low calorie food images. Appetite. Jun 2011;56(3):577–86. doi:10.1016/j.appet.2011.01.029 21291928

[28] Castellanos EH, Charboneau E, Dietrich MS, Obese adults have visual attention bias for food cue images: evidence for altered reward system function. International journal of obesity (2005). Sep 2009;33(9):1063–73. doi:10.1038/ijo.2009.138 19621020

[29] Nijs IMT, Muris P, Euser AS, Franken IHA. Differences in attention to food and food intake between overweight/obese and normal-weight females under conditions of hunger and satiety. Appetite. 2010/04/01/ 2010;54(2):243–254. doi:10.1016/j.appet.2009.11.004 19922752

[30] Flack KD AIRE, McFee KF Kryscio R, Rush CR Exercise Increases Attentional Bias towards Food Cues in Individuals Classified as Overweight to Obese. Physiology & Behavior. 2022;In Press doi:10.1016/j.physbeh.2022.113711 PMC8845497 35066060

[31] Schubert MM, Seay RF, Spain KK, Clarke HE, Taylor JK. Reliability and validity of various laboratory methods of body composition assessment in young adults. Clinical physiology and functional imaging. Mar 2019;39(2):150–159. doi:10.1111/cpf.12550 30325573

[32] Schutte JE, Townsend EJ, Hugg J, Shoup RF, Malina RM, Blomqvist CG. Density of lean body mass is greater in blacks than in whites. J Appl Physiol Respir Environ Exerc Physiol. Jun 1984;56(6):1647–9. doi:10.1152/jappl.1984.56.6.1647 6735823

[33] Siri WE. Body composition from fluid spaces and density: analysis of methods. 1961. Nutrition (Burbank, Los Angeles County, Calif). Sep-Oct 1993;9(5):480–91; discussion 480, 492. 8286893

[34] Epstein LH, Leddy JJ, Temple JL, Faith MS. Food reinforcement and eating: a multilevel analysis. Psychol Bull. Sep 2007;133(5):884–906. doi:10.1037/0033-2909.133.5.884 17723034 PMC2219695

[35] Robinson MJ, Fischer AM, Ahuja A, Lesser EN, Maniates H. Roles of “Wanting” and “Liking” in Motivating Behavior: Gambling, Food, and Drug Addictions. Curr Top Behav Neurosci. 2016;27:105–36. doi:10.1007/7854_2015_387 26407959

[36] Marks KR, Roberts W, Stoops WW, Pike E, Fillmore MT, Rush CR. Fixation time is a sensitive measure of cocaine cue attentional bias. Addiction (Abingdon, England). Sep 2014;109(9):1501–8. doi:10.1111/add.12635 24894879 PMC4612370

[37] Roberts W, Fillmore MT, Milich R. Drinking to distraction: does alcohol increase attentional bias in adults with ADHD? Exp Clin Psychopharmacol. Apr 2012;20(2):107–17. doi:10.1037/a0026379 22121850 PMC3338153

[38] Marks KR, Pike E, Stoops WW, Rush CR. Test-retest reliability of eye tracking during the visual probe task in cocaine-using adults. Drug Alcohol Depend. Dec 1 2014;145:235–7. doi:10.1016/j.drugalcdep.2014.09.784 25456573 PMC4268011

[39] Doolan KJ, Breslin G, Hanna D, Murphy K, Gallagher AM. Visual attention to food cues in obesity: an eye-tracking study. Obesity (Silver Spring). Dec 2014;22(12):2501–7. doi:10.1002/oby.20884 25196826

[40] Hume DJ, Howells FM, Rauch HG, Kroff J, Lambert EV. Electrophysiological indices of visual food cue-reactivity. Differences in obese, overweight and normal weight women. Appetite. Feb 2015;85:126–37. doi:10.1016/j.appet.2014.11.012 25464021

[41] Nijs IM, Muris P, Euser AS, Franken IH. Differences in attention to food and food intake between overweight/obese and normal-weight females under conditions of hunger and satiety. Appetite. Apr 2010;54(2):243–54. doi:10.1016/j.appet.2009.11.004 19922752

[42] Hendrikse JJ, Cachia RL, Kothe EJ, McPhie S, Skouteris H, Hayden MJ. Attentional biases for food cues in overweight and individuals with obesity: a systematic review of the literature. Obes Rev. May 2015;16(5):424–32. doi:10.1111/obr.12265 25752592

[43] Epstein LH, Carr KA. Food reinforcement and habituation to food are processes related to initiation and cessation of eating. Physiol Behav. Oct 1 2021;239doi:ARTN 113512 10.1016/j.physbeh.2021.113512 PMC9632495 34217735

[44] Calitri R, Pothos EM, Tapper K, Brunstrom JM, Rogers PJ. Cognitive biases to healthy and unhealthy food words predict change in BMI. Obesity (Silver Spring). Dec 2010;18(12):2282–7. doi:10.1038/oby.2010.78 20379149

[45] Brand J, Masterson TD, Emond JA, Lansigan R, Gilbert-Diamond D. Measuring attentional bias to food cues in young children using a visual search task: An eye-tracking study. Appetite. May 1 2020;148:104610. doi:10.1016/j.appet.2020.104610 31958480 PMC7995328

[46] Hagan KE, Alasmar A, Exum A, Chinn B, Forbush KT. A systematic review and meta-analysis of attentional bias toward food in individuals with overweight and obesity. Appetite. Aug 1 2020;151:104710. doi:10.1016/j.appet.2020.104710 32298701

[47] Kemps E, Tiggemann M, Hollitt S. Longevity of attentional bias modification effects for food cues in overweight and obese individuals. Psychol Health. Jan 2 2016;31(1):115–129. doi:10.1080/08870446.2015.1077251 26230456

[48] Robinson TE, Berridge KC. The Neural Basis of Drug Craving - an Incentive-Sensitization Theory of Addiction. Brain Res Rev. Sep-Dec 1993;18(3):247–291. doi:Doi 10.1016/0165-0173(93)90013-P 8401595

[49] Jastreboff AM, Sinha R, Lacadie C, Small DM, Sherwin RS, Potenza MN. Neural correlates of stress- and food cue-induced food craving in obesity: association with insulin levels. Diabetes Care. Feb 2013;36(2):394–402. doi:10.2337/dc12-1112 23069840 PMC3554293

[50] Carr KA, Lin H, Fletcher KD, Two functional serotonin polymorphisms moderate the effect of food reinforcement on BMI. Behav Neurosci. Jun 2013;127(3):387–99. doi:10.1037/a0032026 23544600 PMC4049455

[51] Scheid JL, Carr KA, Lin H, FTO polymorphisms moderate the association of food reinforcement with energy intake. Physiol Behav. Jun 10 2014;132:51–6. doi:10.1016/j.physbeh.2014.04.029 24768648 PMC4665647

[52] Flack K, Pankey C, Ufholz K, Johnson L, Roemmich JN. Genetic variations in the dopamine reward system influence exercise reinforcement and tolerance for exercise intensity. Behav Brain Res. Dec 16 2019;375:112148. doi:10.1016/j.bbr.2019.112148 31404557

[53] Epstein LH, Temple JL, Neaderhiser BJ, Salis RJ, Erbe RW, Leddy JJ. Food reinforcement, the dopamine D2 receptor genotype, and energy intake in obese and nonobese humans. Behav Neurosci. Oct 2007;121(5):877–86. doi:10.1037/0735-7044.121.5.877 17907820 PMC2213752

[54] Smith E, Treffiletti A, Bailey PE, Moustafa AA. The effect of attentional bias modification training on food intake in overweight and obese women. J Health Psychol. Sep 2020;25(10–11):1511–1521. doi:10.1177/1359105318758856 29519156

[55] Houben K, Giesen J. Will work less for food: Go/No-Go training decreases the reinforcing value of high-caloric food. Appetite. Nov 1 2018;130:79–83. doi:10.1016/j.appet.2018.08.002 30077731

[56] Stott N, Fox JRE, Williams MO. Attentional bias in eating disorders: A meta-review. Int J Eat Disord. Aug 2021;54(8):1377–1399. doi:10.1002/eat.23560 34081355

